# Assessment of Neutrophil Extracellular Traps (NETs) and Macrophage Ratio in the Inner and Outer Area of Carotid Artery Stenosis

**DOI:** 10.30699/ijp.2022.538238.2713

**Published:** 2022-08-28

**Authors:** Shirin Saberianpour, Mohammad Hadi Saeed Modaghegh, Mohammad Mahdi Kamyar

**Affiliations:** *Vascular and Endovascular Surgery Research Center, Mashhad University of Medical Sciences, Mashhad, Iran*

**Keywords:** Arteries, NETs, Macrophage, Plaque

## Abstract

**Background & Objective:**

It is noteworthy that majority of the data links neutrophil extracellular traps (NETs) to human arterial thrombosis. In the current study, extracellular neutrophil networks and macrophage polarization were assessed in the area outside and inside the Carotid artery stenosis.

**Methods:**

The sample of Carotid plaque of the patient was divided into two halves with a transverse incision; the terms inner part and outer part were used for the plaque's inner part and the adjacent area. Samples were sorted in 10% formalin for CD163, CD11c, MPO, and histone H3 immunohistochemical assessment, while part of the sample was stored at -80°C for western blotting assay for PDA4 marker.

**Results:**

Results of this study showed that the extracellular neutrophil in the inner part of the Carotid plaque was significantly increased (*P*<0.0001), while the number of M1 and M2 macrophages was higher in the inner part compared with the outer part of the Carotid plaque (*P*<0.0001).

**Conclusion:**

The distribution of NETs and the ratio of macrophages may be different in the inner and outer aspects of arterial plaque.

## Introduction

Occlusion of the arteries is a disease that involves plaque formation in the arteries. These plaques consist of fibrous tissue, cholesterol, calcium, and cell debris adhering to the artery wall (1). These plaques are known as atherosclerosis. Such plaques allow blood entrance to the brain, causing a disorder. Due to potential brain damage, artery occlusion is considered a serious disease. The findings of the previous studies suggest that NETs (Neutrophil extracellular traps) are important and significantly influence the development and progression of atherosclerotic plaques (2). In addition, NETs may induce and lead to arterial thrombus formation, stability, and expansion. Neutrophils, macrophages, and less importantly, eosinophils and mast cells produced ETs (Extracellular traps) following the onset of the complications of coronary plaques (3). In turn, ETs formation spans all the steps of the evolution of coronary thrombosis. Various leucocyte types and their ETs are involved in orchestrating the organization and maturation of the thrombus towards stability time‐dependently (4).

More knowledge regarding ETs' roles in athero-thrombotic disease is crucial since it can provide novel strategies to treat cardiovascular diseases. In the case of totally maximum intima‐media thicknesses of the internal arteries, heterogeneous plaques were shown to be associated with the risk of lacunar infarction, stroke, cardiovascular disease, and coronary artery disease (5). The morphology of artery plaques and the internal arteries stenosis degree are mutually dependent factors, showing the severity of the atherosclerotic disease. The immune cells show a higher inflammatory status in the arteries plaque compared to similar cells of the peripheral blood circulation (6). The expression of both anti-inflammatory and pro-thrombotic/proinflammatory mediators are altered in the plaque's milieu suggesting the significant role of balance between these mediators in the progression of artery disease (7). Elevated macrophage density in the atherosclerotic plaques of arteries has been shown to be associated with plaque, lipid content, and elevated plasma levels of low-density lipoprotein cholesterol. Studies have revealed that the atherosclerotic plaque's M1 macrophage content is associated with elevated inflammation or fibrinolysis and the incidence of clinical ischemic stroke (8). The production of NETs was first described as an unrecognized neutrophils' defense mechanism since it was able to entrap and probably eliminate a wide variety of pathogens (8, 9). Yet, mounting evidence has demonstrated that NETs are involved in various pathophysiological conditions. NETs were shown to be present in the atherosclerotic plaques of humans and animal models and are engaged in various mechanisms leading to atherogenesis (10). NETs are among the factors that induce oxidative stress and oxidize high-density lipoprotein particles, thus decreasing their favorable capacity for cholesterol efflux. Also, NETs induce the dysfunction and apoptosis of endothelial cells and enhance the production of anti-ds-DNA autoantibodies (11, 12). In addition, NETs enhance the pro-thrombotic molecules accumulation, including fibrinogen and von Willebrand factor, thus significantly leading to thrombus formation. It is noteworthy that majority of the data links NETs to arterial and venous thrombosis in animal models and humans (13). In the current study, extracellular neutrophil networks and macrophage polarization were assessed in the outside and inside areas of the Carotid plaque.

## Material and Methods

**Sample Collection**

The case study was performed with the approval of the Medical Ethics Committee of Mashhad University of Medical Sciences. According to the symptoms of the disease and diagnostic tests such as angiography, all the referred patients to the hospital had been reported to have carotid plaque. Confirmed casees of carotid plaque were selected. Samples of Carotid plaque of each patient were divided and defined into two halves; the inner part of the plaque is the part adjacent to the vessel's lumen, and the outer part is attached to the wall of the vessel. All the selected patients in this study had no inflammatory or autoimmune diseases and did not take any immunosuppressive drugs. The Carotid plaques were excised, and half of them were stored in 10% formalin, while the other halves were stored at -80°C for molecular studies.

**Immunohistochemical Studies**

The immunohistochemical assay for CD163, CD113, MPO, and Histone H3 marker was done on two groups consisting of both inner and outer parts of the Carotid plaque using the Detection Kite. First, the excised tissues were put in the tissue processor device (Tissue processor, POOYAN model: MIK 2230, serial 223073, made in Iran) before undergoing tissue passage. Then, the tissues were infiltrated using liquid paraffin; then, they were formed into blocks. A microtome was used to cut the blocks; the produced sections were put on glass slides. Following the preparation, fixation, and molding of the tissue, the slides of the samples were prepared. Deparaffinized slides were done with added peroxidase inhibitor in the dark for ten minutes to inhibit the endogenous peroxidase. Ten minutes later, the slides were rinsed using PBS buffer three times. Then, the prepared solutions comprised primary antibodies for Mouse anti-human CD163 Monoclonal Antibody (Clone MRQ-26), Anti-CD11c antibody [N418] (ab33483), Anti-Histone H3 antibody (ab18521), Anti-Myeloperoxidase antibody (ab9535), were incubated at room temperature for 2-3 hours. Then, the PBS buffer was rinsed three times. Master Polymer Plus Detection System Peroxidase (100 microliters) was added to any of the samples and then rinsed three times following incubation for 30 minutes at room temperature. The prepared slides were investigated using a light microscope.

**Investigation of PAD4 Protein Expression using Western Blotting**

The PAD4 (Peptidyl arginine deiminase 4) level was measured using western blotting; protein lysate (50 μg) was placed in each lane and electrophoresed in SDS-PAGE (10%) and transferred to the membrane of polyvinylidene di-ﬂuoride (Merck Millipore). A primary antibody was used against PAD4 (Cell Signaling) for the immune reaction. In brief, bovine serum albumin 1% (Sigma) was used to block the membranes for 1 hour; then, the membranes were incubated using the diluted antibody (with 1: 1000 dilution), which contained bovine serum albumin (5% w/v), Tween 20 (0.1%), and 1X Tris-buﬀered saline, overnight. After rinsing phosphate buﬀered saline and 0.1% Tween 20 (10 minutes each) three times, the membranes were incubated using HRP-conjugated secondary antibodies (Cat no:7074; Cell Signaling) at RT for 1 hour. Then, the membranes were rinsed three times with phosphate buﬀered saline. X-ray films and the ECL system (Roche) were used to visualize the immune-reactive bands. Band densities were calculated using ImageJ software (ver. 1.4). This assay was done in triplicate. The relative PAD4 peptide content was expressed after normalization to the GAPDH housekeeping protein (Abcam).

**Statistical Analysis**

We used Graph Pad Prism 8 to perform our *statistical* calculations. For statistical analysis, we used independent samples T-test or its non-parametric equivalents. Mann-Whitney U test was used for quantitative variables and Chi-square (or Fisher's exact test) for qualitative variables.

## Results

**Confirmation of Carotid Plaque in Patient**

**Fig. 1:**
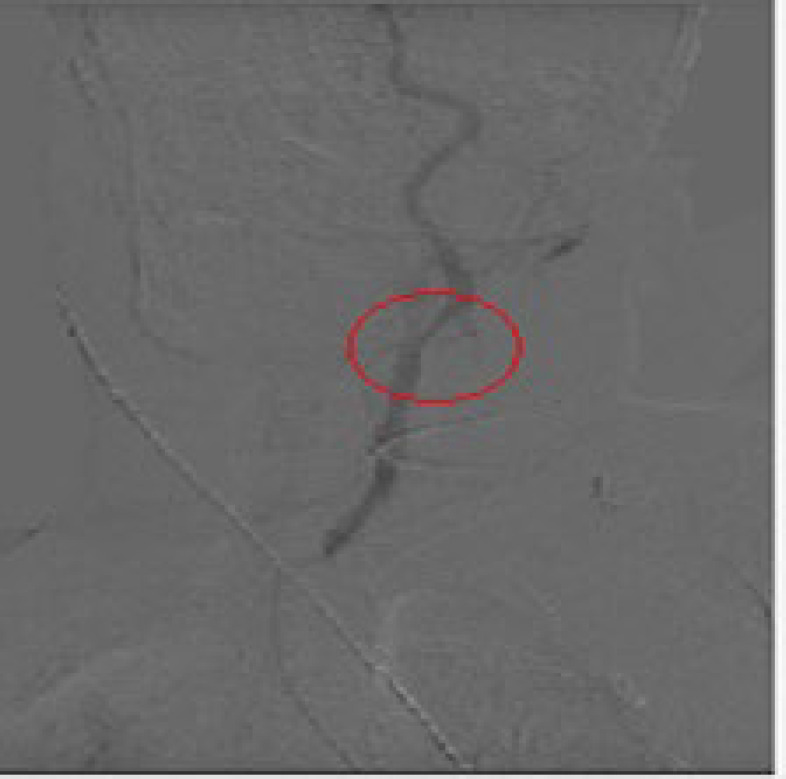
The angiographic image of the patient with Carotid Artery Stenosis; Carotid plaque is located between the internal carotid artery and common carotid artery

Physical examination and clinical history were the first steps for diagnosis the disease. Carotid plaque can be suspected by listening to the sound of arteries in the neck area with a medical earphone. The presence of sound in this area is one of the characteristics of Carotid stenosis. Next, the patient's physical and mental abilities (such as strength, memory, and speech) should be examined. If carotid stenosis is suspected, the doctor prescribes one of the diagnostic angiographic tests. In Figure 1, contrast injection shows carotid stenosis in angiographic images.

**Increase in M1 and M2 Macrophages in Outer Area compared to Inner Area**

**Fig. 2:**
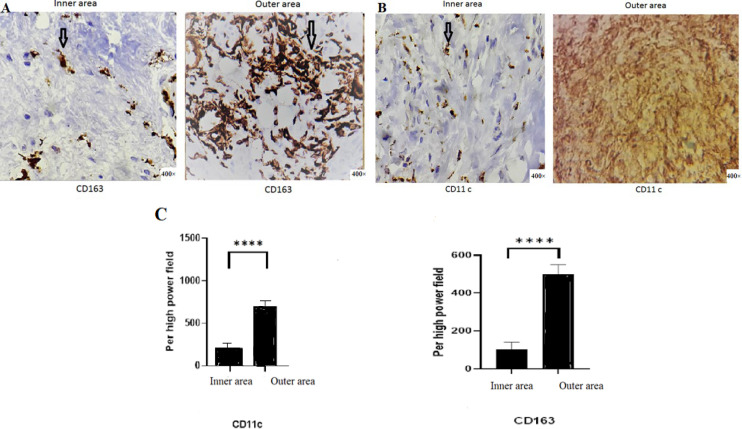
Immunohistochemical staining for CD163 and CD11c from the inner area toward the outer area of the Carotid artery plaque

**Fig. 3:**
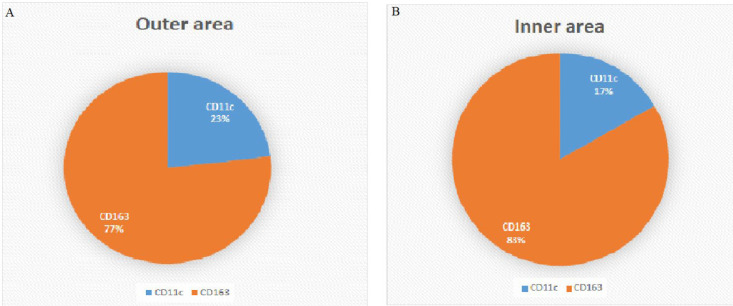
M1 (CD11c) to M2 (CD163) ratio in the outer and inner area of arteries plaque; A: 23% CD11c, 77%CD163 in the outer area of artery plaque B: 17%CD11c and 83%CD163 in the inner area of the Carotid artery plaque

The CD163 marker is known as the type 2 macrophage marker. The CD11c marker is the type 1 macrophage (*P*<0.0001). On the other hand, the ratio of M1 to M2 macrophages in the inner region showed an increase from 0.2 to 0.3. In contrast, the ratio of M2 to M1 macrophages showed a decrease from 5 in the inner region to 3.3 in the outer region (Figure 2 and Figure 3).

A; Microscopic photos of immunohistochemical staining for CD163 from the inner area toward the outer area of the Carotid artery plaque 400× magnification)

B; Microscopic photos of Immunohistochemical staining for CD11c from the inner area toward the outer area of the Carotid artery plaque (400× magnification)

C: Increased CD11c expression in the outer area compared to the inner area of the Carotid artery plaque (*P*<0.0001 ****), Increased CD163 expression in the outer area compared to the inner area of the Carotid artery plaque (*P*<0.0001 ****).

**Increased Extracellular Network of Neutrophils in the Inner Region of the Arteries Plaque Compared to the Outer Region of the Carotid Artery Plaque**

**Fig. 4:**
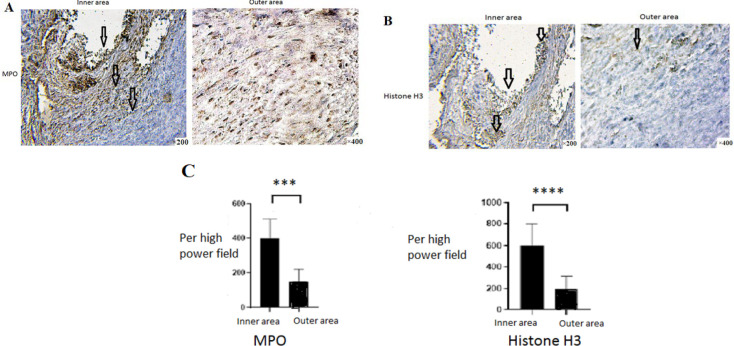
Assessment of NETs in the outer and inner area of the Carotid artery plaque by Immunohistochemical method

MPO (Myeloperoxidase) MPO showed high expression of neutrophil granulocytes. The immunohistochemical results of the Carotid plaque tissues showed that the expression of MPO in the inner region of the Carotid plaque a significant increase compared to the outer region (*P*<0.001). Histone 3 H3 In the inner of the Carotid plaque was significantly increased compared to the outer region (*P*<0.0001). It showed an increase in the extracellular networks of neutrophils, which demonstrated a visible increase in the inner region of the Carotid plaque compared to the outer region (Figure 4).

**Increased Expression of PAD4 Protein in the Inner Region of the Tumor Compared to the Outer Region**

**Fig. 5:**
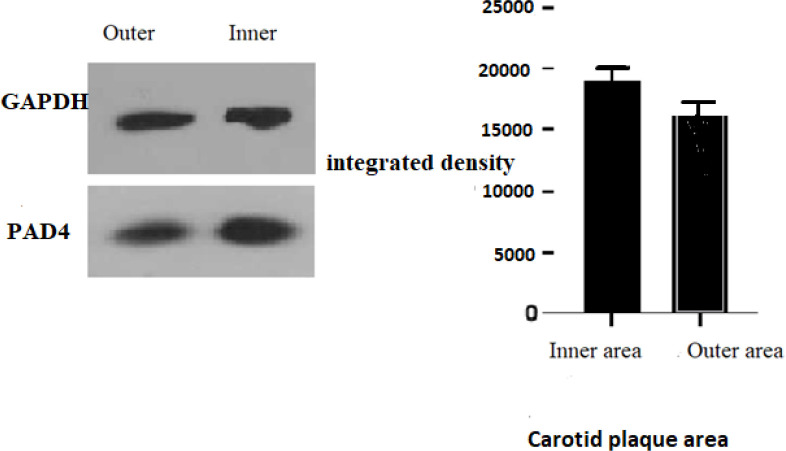
The western blot technique detected the level of PAD4 in the inner and outer areas of Carotid artery plaque

Peptidyl arginine deiminase-4 (PAD4) is indispensable for generating neutrophil extracellular traps (NETs). Western blotting test showed an increase in the level of PAD4 in the inner area compared to the outer area, but there was no statistically significant difference (Figure 5).

A; Microscopic photos of immunohistochemical staining for MPO from the inner area toward the outer area of the Carotid artery plaque (200× and 400× magnification)

B; Microscopic photos of immunohistochemical staining for histone H3 from the inner area toward the outer area of the Carotid artery plaque (200× and 400× magnification)

C; Increased MPO expression in the inner area compared to the outer area of the Carotid artery plaque (*P*<0.001 ***). Increased histone H3 expression in the inner area compared to the outer area of the Carotid plaque (*P*<0.0001****).

## Discussion

Results of this study showed that the extracellular neutrophil traps in the inner part of the arteries plaque were significantly increased (*P*<0.0001), while the number of M1 and M2 macrophages was higher in the inner part compared with the outer part of the arterial plaque (*P*<0.0001).

In the early stage of coronary atherosclerosis in humans, fatty streaks are developed with extracellular lipid deposition, which is associated with specific proteoglycans in the outer part of established diffuse intimal thickening. When the lipid content of the fatty streaks increases, macrophages are infiltrated toward these lipid deposits to form the intimal thickening as foam cells (14).

Previous studies have shown that M1 macrophages are predominant in the rupture-prone shoulder parts, while M2 macrophages are predominant in the adventitia. In the atherosclerotic lesions in humans, M2 macrophages are more commonly found in stable cell-rich areas rather than away from the lipid core (15). Comparing human arteries and femoral atherosclerotic plaques have shown an increase in number of M1 macrophages in the arterial lesions, while M2 macrophages are predominant in the femoral lesions, which indicates that the accumulation of M1 macrophages might be characteristic for symptomatic lesions (16). J. Lauran Stöger* et al.*'s histopathological analysis suggested that M1 and M2 macrophages continuously accumulate in the plaques as the lesion severity progresses (17). In the plaque, as a rupture-prone part of the intima, the pro-thermogenic M1 macrophages are the predominant macrophages, though such a predominance is not observed in the fibrous cap regions (17, 18). Remarkably, the macrophages in vascular adventitia are predominantly of M2 phenotype. Thus, both M1 and M2 subtypes of macrophages characterize different stages of plaque development in humans, though they localize with distinct lesion morphological features (19, 20).

Investigating the potential causes for macrophage dispersion in different parts of the palaques of the arteries Possibly, an effective factor may be wall shear stress, i.e., a frictional force exerted parallel to the vascular wall leading to the altered endothelial cell signaling, endothelial phenotype, gene, and protein expression, which leads to a proinflammatory phenotype, reduces the nitric oxide availability and disrupts the extracellular matrix, resulting in plaque development (21, 22). Emerging experimental and clinical data suggests the pathobiology associated with the abnormal wall shear stress leads to the development and progression of atherosclerotic plaques (23). Mat J. Daemen* et al.*'s study showed that plaque fissures are commonly found in the advanced arteries plaques, which have a grossly normal luminal surface and are associated with fresh hemorrhage in the plaque (24).

It has been shown that neutrophil extracellular traps stimulate the activation of antigen-presenting cells, endothelial cells, and platelets, which leads to a proinflammatory immune response (25). Generally, this finding suggests their presence in the plaques and thrombi and their potentially causative role in promoting the formation of atherosclerotic plaque and arterial thrombosis (25). 

Studies suggest the higher usability of the outer layers of the arteries plaques is lower than the inner layers; thus, parts of the plaques may separate and enter the peripheral blood circulation and migrate to the other body parts (26). Also, the inner layer of the arteries plaque plays a key role in plaque development, so the extracellular network accumulation in this area potentially effectively prompts platelet and lipid accumulation and increases the plaque size (27).

Knight* et al.*'s study showed that NET formation might be prevented using chloramine treatment which inhibits PAD4 (playing an important role in NET formation 32), thereby reducing the size of the atherosclerotic plaque and arteries artery thrombosis postponed in the mouse model of atherosclerosis (28, 29).

NETs have great potential for further investigation to find a better treatment for venous thromboembolism and atherosclerosis plaques. Therefore, developing drugs to target extracellular networks, including NETs, in addition to macrophage anti-polarization therapies to M1, which form the plaque cores, may weaken the plaque formation primarily and prevent further plaque expansion and carotid plaque occlusion (30).

## Conclusion

The distribution of NETs and the ratio of macrophages are different in the inner and outer parts of the carotid plaque. 

## Conflict of Interest

The authors declared no conflict of interest.
